# A Reliable Energy-Efficient Multi-Level Routing Algorithm for Wireless Sensor Networks Using Fuzzy Petri Nets

**DOI:** 10.3390/s110303381

**Published:** 2011-03-22

**Authors:** Zhenhua Yu, Xiao Fu, Yuanli Cai, Mehmet C. Vuran

**Affiliations:** 1 School of Telecommunication Engineering, Air Force Engineering University, Xi’an 710077, China; E-Mail: fuxiao@126.com; 2 School of Electronic and Information Engineering, Xi’an Jiaotong University, Xi’an 710049, China; E-Mail: ylicai@mail.xjtu.edu.cn; 3 Cyber-Physical Networking Laboratory, Department of Computer Science and Engineering, University of Nebraska-Lincoln, Lincoln, NE 68588, USA; E-Mail: mcvuran@cse.unl.edu

**Keywords:** wireless sensor networks, routing algorithm, clustering, fuzzy Petri nets

## Abstract

A reliable energy-efficient multi-level routing algorithm in wireless sensor networks is proposed. The proposed algorithm considers the residual energy, number of the neighbors and centrality of each node for cluster formation, which is critical for well-balanced energy dissipation of the network. In the algorithm, a knowledge-based inference approach using fuzzy Petri nets is employed to select cluster heads, and then the fuzzy reasoning mechanism is used to compute the degree of reliability in the route sprouting tree from cluster heads to the base station. Finally, the most reliable route among the cluster heads can be constructed. The algorithm not only balances the energy load of each node but also provides global reliability for the whole network. Simulation results demonstrate that the proposed algorithm effectively prolongs the network lifetime and reduces the energy consumption.

## 1. Introduction

Wireless sensor networks (WSNs) have become a vibrant and exciting research and development area in recent years and can be used in many different applications, including battlefield surveillance, home security, smart spaces, environmental monitoring, and target tracking [1,2].

A wireless sensor network consists of a large number of tiny, low-powered, energy-constrained sensor nodes with sensing, data processing and wireless communication components. Sensor nodes in WSNs are small battery powered devices with limited energy resources, and their batteries cannot be recharged once the sensor nodes are deployed. Therefore, minimizing energy consumption is an important issue in the design of WSNs protocols. The energy is also the major consideration in designing the routing protocol. Cluster routing is an effective solution in reducing energy consumption, prolonging the lifetime of the networks and providing network scalability [3]. In the cluster routing, sensor nodes are divided into groups and form clusters. Each cluster has a leader, often referred to as the cluster head [4]. A cluster head can collect data from nodes within the cluster, and aggregate the data and forward it to the base station. Through data aggregation of the sensors’ data at the cluster heads, the total amount of data sent to the base station can be significantly decreased. Therefore the overhead can be significantly reduced, and energy and bandwidth resources can be saved.

In recent years clustering routing technique has been widely investigated in the context of WSNs [4]. Although the existing cluster-based routing protocols have been proposed, most of them focus on the node energy and the number of hops in a cluster instead of the network topology and reliability of routes [5]. The route with the minimum number of hops may not always be the optimal and reliable route. Since WSNs suffer from different kinds of uncertainty, randomness, and fuzziness [6], a reliable routing protocol with adapting capabilities to high variability and uncertainty is needed. In this paper, we aim to enhance the energy efficiency of WSNs, improve the route reliability, and balance the energy consumption among sensor nodes. To this end, a reliable energy-efficient multi-level routing algorithm for wireless sensor networks (REEMR) is presented, where fuzzy Petri nets are introduced to overcome the above shortcomings. REEMR parameters can be determined more accurately and dynamically by fuzzy reasoning using fuzzy Petri nets. Experimental results demonstrate that the presented algorithm can provide significant energy savings while achieving reasonably high performance for WSNs.

Our main contributions include

1. Fuzzy production rules are mapped into fuzzy Petri nets for representation of a network topology.
2. A cluster head selection mechanism is developed. This mechanism considers the residual energy, number of neighbors, and centrality of each node and uses fuzzy Petri nets for cluster heads selection.
3. Using fuzzy Petri nets and a reasoning mechanism, a reliable multi-hop routing algorithm, which creates routes among cluster heads, is developed.
4. The resulting reliable energy-efficient multi-level routing (REEMR) protocol is implemented and evaluated through simulations.

The remaining of the paper is organized as follows: In Section 2 we review the relevant work. In Section 3, background on fuzzy Petri nets for the representation of network topology is provided. In Section 4, the REEMR protocol, which includes a clustering algorithm and a reliable routing algorithm among cluster nodes, is presented. In Section 5, we evaluate the performance of REEMR protocol and compare it to several protocols. Finally, Section 6 concludes the paper.

## 2. Related Work

The cluster routing technique involves sensor nodes in multi-hop communication within a cluster, and then the cluster head aggregates the data to decrease the number of transmitted messages to the base station.

Low-energy adaptive clustering hierarchy (LEACH) [7] is the first cluster-based routing protocols in wireless sensor networks. LEACH selects cluster heads with some probability, and the cluster heads fuse and aggregate data arriving from nodes that belong to the respective cluster. Cluster heads are periodically rotated among the nodes to balance energy consumption, and enhances the network lifetime. However, some cluster heads may be very close to each other and cannot be uniformly deployed in the networks by probability mechanism, and cluster heads number is not always equal to the preestablished number. To uniformly deploy cluster heads, a centralized version of LEACH, LEACH-C [3], and a centralized energy-efficient routing protocol–BCDCP [8] are proposed. However, these centralized algorithms bring worse scalability and robustness to large networks than distributed algorithms.

To overcome the limitations of LEACH, a fuzzy logic approach to cluster head election [9] is proposed which uses three fuzzy variables (concentration, energy and centrality). However, this algorithm is a centralized election mechanism, and the base station has to collect the energy and distance information from all sensor nodes. In [10], cluster head election mechanism using fuzzy logic (CHEF) is proposed, which is a localized cluster head election mechanism. CHEF uses energy and local distance as fuzzy variables in the fuzzy if-then rules. Simulation results show that the cluster heads in CHEF are more evenly distributed over the network than those in LEACH, and then CHEF further prolongs the network lifetime. But CHEF does not construct multi-hop routes in cluster heads.

A generalized fuzzy logic based energy-aware routing [11] is presented which is a soft, tuneable parameter based algorithm. But this algorithm assumes that a cluster head is much powerful as compared to the other sensor nodes and has no energy limitation. A fuzzy self-clustering algorithm (FSCA) [12] considers the node residual energy and local density to improve the lifetime of WSNs. To uniformly distribute clusters over the networks, FSCA employs migration fuzzy module to recluster and merge existed clusters. However, reclustering the whole network adds more control overhead and needs more time. In [13], an energy and mobility-aware geographical multipath routing (EM-GMR) algorithm is presented, which is based on fuzzy logic system considering the remaining battery capacity, mobility, and distance to the destination node.

Power-efficient gathering in sensor information systems (PEGASIS) [14] organizes sensor nodes into a chain using a greedy algorithm, so each node only communicates with its neighbors. Nevertheless, PEGASIS requires the global knowledge of the network topology and the farther nodes will result in a bigger data delay. A hybrid energy-efficient distributed clustering approach (HEED) [15] uses a hybrid metric consisting of residual energy and communication cost as attributes for cluster head election. The primary parameter is residual energy and the secondary parameter is the average minimum reachability power. HEED ensures a uniform distribution of cluster heads across the network and adjusts the probability of cluster head election to ensure intracluster connectivity. However, HEED relies on the assumption that cluster heads can communicate with each other and form a connected graph; it is difficult to realize HEED in practical WSNs.

In a distributed energy-efficient clustering algorithm (DEEC) [16], cluster heads are elected by a probability based on the ratio between the residual energy of each node and the average energy of the total networks. However, DEEC is required to estimate the total energy of the networks according to the network topology and broadcasts it to all nodes. Realizing this in practical deployments could be tricky. A new energy-efficient clustering approach (EECS) [17] for single-hop wireless sensor networks is presented which is more suitable for the periodical data gathering applications. EECS extends LEACH algorithm by dynamic sizing of clusters based on cluster distance from the base station. However, EECS does not consider the structural characteristics of network topology and thus cluster heads are only elected on the basis of residual energy.

Energy-efficient cluster formation protocol (EECF) [18] selects cluster heads following a three-way message exchange between each sensor and its neighbors. Cluster head election is mainly based on sensors’ respective residual energies and degrees. The message exchange complexity of EECF is *O*(1) and a worst-case convergence time complexity is *O*(*N*). In [19], a novel energy efficient cluster formation algorithm based on a multi-criterion optimization technique is presented, which uses multiple individual metrics in the cluster head selection process as input while simultaneously optimizing on the energy efficiency of the individual sensor nodes as well as the overall system. However, the multi-criterion optimization technique involves some complex matrix operations. As sensor nodes are significantly constrained in computational capacity, it is difficult for them to execute complex matrix operations.

## 3. Background on Fuzzy Petri Nets

Fuzzy Petri nets (FPN) [20] can be used to represent structured knowledge and describe the procedure for supporting fuzzy reasoning automatically in a rule-based expert system. A generalized fuzzy Petri net is defined as a 10-tuple,

(1) F P N=(P, T, I, O, D, W, μ, f, α, β)

where

*P* = {*p*_1_, *p*_2_, . . ., *p_n_*} denotes a finite nonempty set of places;
*T* = {*t*_1_, *t*_2_, . . ., *t_m_*} denotes a finite nonempty set of transitions;
*I* : *T* → *P*^∞^ is the input function, a mapping from transitions to bags of places;
*O* : *P* → *T*^∞^ is the output function, a mapping from places to transitions;
*D* = {*d*_1_, *d*_2_, . . ., *d_n_*} denotes a finite set of propositions, |*P*| = |*D*|;
*W* : *I* → [0, 1] denotes the weights of the input functions, the sum of the weights *W* in the input arcs of a transition is 1, which denotes the influence strength of the input to the output.
*μ* : *T* → [0, 1] denotes the certainty factors of fuzzy rules;
*f* : *T* → [0, 1] denotes the threshold of a transition firing;
*α* : *P* → [0, 1] is an association function which maps from places to real values between zero and one;
*β* : *P* → *D* is also an association function mapping from places to propositions.

Fuzzy Petri nets are different from ordinary Petri nets due to the features of fuzzy production rule systems that are different from discrete event systems [21]. In FPN, the number of tokens in a place cannot be greater than one. Furthermore, a token is associated with a truth degree between zero and one. A token does not represent a “resource”, whereas it may likely do so in ordinary Petri nets.

The enabling and firing rules in FPN are given as follows:

1. A transition *t* ∈ *T* is said to be enabled if ∀*p* ∈^•^ *t* : *M*(*p*) = 1 ∧ *α*(*p*) × *W* ≥ *f*, denoted as *M*[*t* >.
2. Enabled at marking *M*, *t* may fire resulting in a new marking *M′*, denoted as *M*[*t* > *M′*, and that
  $$M\prime \left(p\right)=\{\begin{array}{ccc} M\left(p\right) & : & p\in^{•}t\backslash t^{•}\\ F\left(M\left(p\right)+\alpha \left(p\right)\times W\times \mu \left(t\right)\right) & : & p\in t^{•}\backslash^{•}t\\ M\left(p\right) & : & \text{otherwise} \end{array}$$
  where
  $$F(x)=\{\begin{array}{lll} x & \text{if} & 0<x<1\\ 1 & \text{if} & x\geq 1 \end{array}$$

Fuzzy Petri nets may depict the fuzzy relationships between many propositions. Suppose a set *R* of fuzzy production rules, *R* = {*r*_1_, *r*_2_, . . ., *r_n_*}, the *i*th fuzzy production rule is given as follows:

(2) $$R_{i}\;:\;\text{IF}\;\;d_{j}\;\text{THEN}\;\;d_{k}\;\;(C\;F=\mu_{i})\;\;(i=1,2,\ldots ,n)$$

where *d_j_* and *d_k_* are propositions with some fuzzy variables. The certainty factor (CF) is a value between zero and one, *μ ∈* [0, 1], which represents the strength of the certainty in the fuzzy production rule.

**Figure 1.**
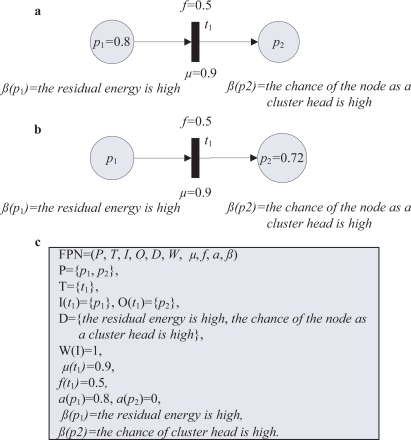
An example fuzzy Petri nets model for network knowledge representation. **(a)** Before firing transition; **(b)** after firing transition; **(c)** fuzzy Petri nets representation.

The fuzzy Petri net model for Equation (2) is shown in Figure 1. Assuming the truth degree of the proposition “the residual energy is high” is 0.8, the transition *t*_1_ fires from its input place (*p*_1_) into an output place (*p*_2_). The certainty factor is 0.9. The truth degree value in the output place (*p*_2_) of *t*_1_ is calculated as 0.72 (by 0.8× 0.9) when *t*_1_ fires. According to the value, the node with higher residual energy may be elected as the cluster head.

**Figure 2.**
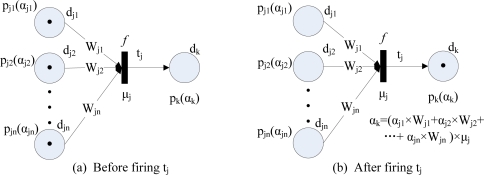
Fuzzy Petri nets representation of type 1 rules.

**Figure 3.**
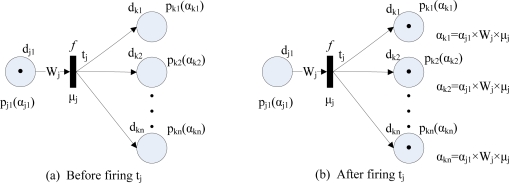
Fuzzy Petri nets representation of type 2 rules.

**Figure 4.**
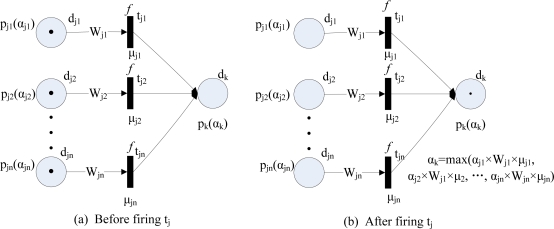
Fuzzy Petri nets representation of type 3 rules.

A composite fuzzy production rule denotes its antecedent or consequence portion contains AND or OR connector. The composite fuzzy production rules can be classified into the following types:

- **Type** 1: IF *d*_*j*1_ AND *d*_*j*2_ AND . . . AND *d_jn_* THEN *d_k_* (CF=*μ_j_*). This rule type can be modelled by FPN as shown in Figure 2(a), and its fuzzy reasoning process is shown in Figure 2(b).
- **Type** 2: IF *d_j_* THEN *d*_*k*1_ AND *d*_*k*2_ AND . . . AND *d_kn_* (CF=*μ_j_*). This rule type can be modelled by FPN as shown in Figure 3(a), and its fuzzy reasoning process is shown in Figure 3(b).
- **Type** 3: IF *d*_*j*1_ OR *d*_*j*2_ OR . . . OR *d_jn_* THEN *d_k_* (CF=*μ_j_*). This rule type can be modelled by FPN as shown in Figure 4(a), and its fuzzy reasoning process is shown in Figure 4(b).
- **Type** 4: IF *d_j_* THEN *d*_*k*1_ OR *d*_*k*2_ OR . . . OR *d_kn_* (CF=*μ_j_*). This rule type is unsuitable for deducing control because they do not make specific implication. Therefore, this rule is not allowed to appear in a knowledge base [20].

In the following, the first three types of rules are mainly employed, and the different fuzzy variables are combined into a composite fuzzy production rule.

## 4. REEMR: Reliable Energy-Efficient Multi-Level Routing Algorithm

REEMR consists of a clustering algorithm and a reliable multi-hop routing algorithm, which are employed to divide sensor nodes into clusters and construct a reliable route for cluster heads, respectively.

### 4.1. Fuzzy Logic Rules for Clustering

To balance energy consumption of nodes, REEMR constructs clusters at each round similar to LEACH. To get a cluster head election chance, three fuzzy sets and different fuzzy production rules for knowledge representation are considered. The fuzzy variables that are used in the fuzzy production rules are defined as follows.

1. Residual Energy: The remaining energy of each node. The more residual energy the node has, the more data is processed and transmitted, and the longer lifetime of the node is.
2. Number of Neighbors: The number of neighbor nodes of each node. The number of neighbors affects in some way for proper cluster head election. It is more reasonable to select a cluster head in a region where the node has more neighbors.
3. Centrality: The sum of the distances between the node and its neighbors represents how central the node is to the cluster. The more central the node is to a cluster head, the more is the energy efficiency for it to transmit the data through the cluster head.

The node centrality is the sum of distances between a node and its neighbor nodes. The above fuzzy variables are input fuzzy variables for the fuzzy production rules, and the output variable is the node’s cluster head election chance. The trapezoid functions are employed as the membership functions corresponding to the fuzzy linguistic variables [22], which can be defined as follows.

**Figure 5.**
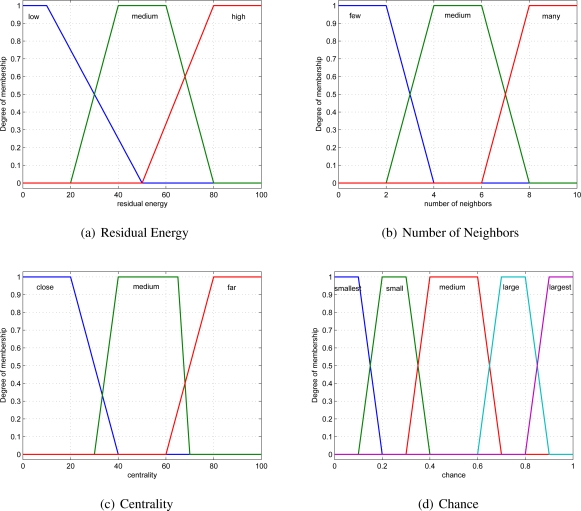
Membership functions of the fuzzy variables.

- The fuzzy variable “residual energy” has three fuzzy sets—high, medium and low, and its membership function is shown in Figure 5(a).
- The fuzzy variable “number of neighbors” has three fuzzy sets—many, medium and few. The possible fuzzy quantization of the range [1,10] for the number of neighbors is an experimental result [23]. The membership function is shown in Figure 5(b).
- The fuzzy variable “centrality” has three fuzzy sets—far, medium and close, and its membership function is shown in Figure 5(c).
- The outcome to represent the node’s cluster head election chance has five fuzzy sets—smallest, small, medium, large, and largest, and its membership function is shown in Figure 5(d).

These membership functions can be set according to the practical need. To calculate a chance to be a cluster head, a typical form of fuzzy production rules for cluster head election is exemplified as follows.

**IF** *the residual energy is high* **AND** *the number of neighbors is few* **AND** *the centrality is close* **THEN** *the node’s cluster head election chance is largest.*

**Figure 6.**
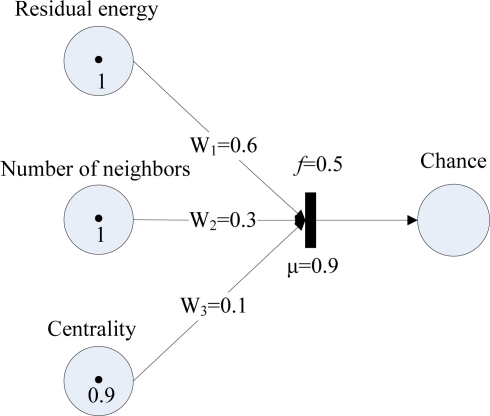
An example fuzzy Petri nets model for a cluster head election rule.

Since each input variable has 3 linguistic states, the total number of possible fuzzy inference rules is 3 × 3 × 3 = 27. According to the composite fuzzy production rule Type 1 in Section 3, a typical fuzzy Petri net model for the above fuzzy production rule is shown in Figure 6, where “residual energy”, “number of neighbors”, and “centrality” are input fuzzy variables, *W*_1_, *W*_2_, *W*_3_ are weights, and the node’s cluster head election chance is the output fuzzy variable. Assuming the truth degrees of the proposition “the residual energy is very high”, the proposition “the number of neighbors is few” and the proposition “the centrality is close” are 1, 1 and 0.9, respectively. Assuming the weights of the arcs are 0.6, 0.3 and 0.1, the threshold is 0.5, and the certainty factor is 0.9. When the transition *t* is enabled and fires, the truth degree value is calculated as *α*(*p*_4_) = (1 × 0.6 + 1 × 0.3 + 0.9 × 0.1) × 0.9 = 0.891.

From the fuzzy Petri net model, each node can get the fuzzy variable *chance*. According to the cluster head election chance *C_i_*, we can get the time *t_i_* of the *i*th node broadcasting the cluster declaring message ADV_Head, as shown in the following.

(3) $$t_{i}=C_{i}\times T$$

where *T* is predefined as the maximum time of cluster heads competing.

If a sensor node does not receive the message ADV_Head before the time *t* is expired, it will broadcast the message ADV_Head to its neighbor nodes. If the *j*th node receives some message ADV_Head before the time *t_j_* is expired, then it will give up the competition for the cluster head, and construct a cluster head candidate table including the nodes which transmit ADV_Head. Finally, the *j*th node selects the node with the maximum chance as its cluster head. If there are multiple nodes having maximum chance, then the node having more energy is selected as the cluster head. Finally, the node transmits the message JOIN to the cluster head.

The clustering algorithm:

**INPUT**: the cluster head election chance *C_i_* of each node *N_i_*.
**OUTPUT**: the set of cluster heads.
**Step 1** Set the timer *t_i_* of node *N_i_* for competing a cluster head and start.
**Step 2** while(the timer *t_i_* is not expired)
  if (*N_i_* does not receive ADV_Head)
    *N_i_* broadcasts ADV_Head to its neighbors
  else if (*N_i_* receive one ADV_Head from the node *N_j_*)
    *N_i_* selects *N_j_* as its cluster head
    *N_i_* transmits the message JOIN to *N_j_*
  else if (*N_i_* receive *k* ADV_Head from other *k* nodes)
    Among the *k* nodes, *N_i_* select the node *N_l_* with the maximum cluster head election chance as its cluster head
    *N_i_* transmits the message JOIN to *N_l_*
  endif
endwhile

### 4.2. Fuzzy Petri Net Based Multi-Hop Routing

After the clusters are formed, cluster heads aggregate the data traffic and forward it to the base station through a multi-hop route. Consequently, energy efficient and robust multi-hop routes among cluster heads should be constructed. As cluster heads change frequently, in this paper, we use fuzzy Petri nets and the reasoning mechanism to propose a reliable multi-hop routing among cluster heads.

**Figure 7.**
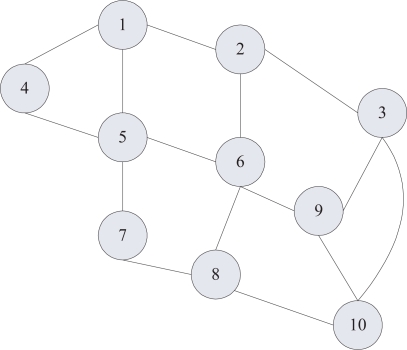
Topology of cluster heads.

**Figure 8.**
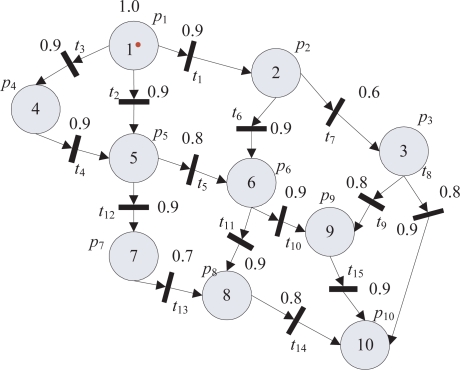
FPN model of topology of cluster heads.

In Figure 7, the network topology of cluster heads, where the node 10 represents a base station, is shown. According to the definition of FPN, a place *p* represents a cluster head, *α*(*p*) denotes the truth degree of reliability, a transition *t* is set up between the neighboring cluster heads, and then places and their corresponding transitions are connected using arcs. One-way direction from the source node to the destination node is chosen, and then FPN model of the network topology is shown in Figure 8. The initial mark of *p*_1_ is 1.0.

Certainty factor and threshold value of every transition in the FPN model evaluate the reliability between each neighboring cluster head. The higher the certainty factor, the more reliable the link between two cluster heads. If the degree of reliability *α*(*p_i_*) of place *p_i_* exceeds the corresponding threshold value *f*(*t*), the transition *t* will be fired, which means that cluster head *p_i_* can communicate with the neighboring cluster head *p_j_*, *α*(*p_j_*) = *α*(*p_i_*) *× μ*(*t*). The threshold should be set according to the specific need. The higher the threshold, the less the links between the neighboring cluster heads. In this paper, to simplify the FPN model, all the thresholds of transitions are unified to be 0.1 and the certainty factor of transition *t* is defined as follows:

(4) $$\mu \left(t\right)=1-0.5\times \frac{d}{R}$$

where *d* is the distance between two neighboring cluster heads, and *R* is the communication radius of cluster heads.

According to Figure 8, when the cluster head *p*_1_ needs to communicate with the base station *p*_10_, a reliable route needs to be constructed. This problem can be solved by developing a fuzzy reasoning algorithm based on fuzzy Petri net model. Assume that the truth degree of the cluster head *p*_1_ is given. The places *p*_1_ and *p*_10_ are called the starting place and the goal place, respectively. If the truth degree of *p*_1_ is greater than the corresponding threshold of its transition, it will flood a route request (RREQ) packet to its IRS. Otherwise, it will not flood the RREQ for the reason that this route is not reliable for a short time, which reduces the control overhead and provides a more reliable route. If a cluster head receives a RREQ packet from its neighbor and it is not the goal place, it will record the neighbor ID and forward the packet to all of its neighbors. Therefore, after a while, some relay cluster heads will receive at least one RREQ packet sent from the starting place. If the RREQ packets arrive at the goal place *p*_10_, *p*_10_ chooses the route with the largest degree of reliability as its the most reliable route. Finally, *p*_10_ will reply a Reply packet of the most reliable route to the starting place *p*_1_. The Reply control packet travels along the reverse path of the RREQ packet. Then multi-hop route among places is constructed.

The fuzzy reasoning algorithm can be expressed as a sprouting tree [20], where each leaf node is denoted as (*p_k_*, *α*(*p_k_*)). The proposed algorithm is presented as follows:

**INPUT**: the truth degree *α*(*p_s_*) of the proposition *β*(*p_s_*) for the starting place *p_s_*.
**OUTPUT**: the truth degree *α*(*p_j_*) of the proposition for the goal place.
comptruth(*α*(*p_s_*))
begin
  if(IRS(*p_s_*))= ϕ
    exit
  else
    for all *p_k_* ∈IRS(*p_s_*)
      if *α*(*p_s_*) ≥ *f*
        a transition is enabled,
        create a new leaf node (*p_k_*, *α*(*p_k_*)) in the tree,
        and create a new branch labelled *μ* is directed from (*p_s_*, *α*(*p_s_*))
        to (*p_k_, α*(*p_k_*)), where *α*(*p_k_*) = *α*(*p_s_*) × *μ*.
        if (*p_k_* = *p_j_*)
          *p_k_* is called success leaf node
        else
          suppose *p_s_* = *p_k_*
          call comptruth(*α*(*p_s_*))
        endif
      endif
    endfor
  endif
end

The branch from *p_s_* to *p_j_* is called a reasoning path, namely a route from the source cluster head to the base station. Let *Q* be a set of success leaf nodes, *Q* = {(*p_j_*, *α*_1_), (*p_j_*, *α*_2_), . . ., (*p_j_*, *α_l_*)}, where *α_l_* *∈* [0, 1]. Set *z* = Max(*α*_1_, *α*_2_, . . ., *α_l_*). The truth degree for the goal place *p_j_* is *z*.

**Figure 9.**
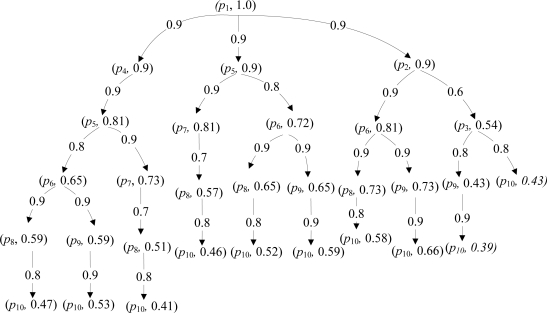
The sprouting tree.

Consider the topology in Figure 8. To choose a reliable route, cluster head *p*_1_ broadcasts a RREQ packet to its neighbors *p*_2_, *p*_4_, and *p*_5_, until the base station receives the RREQ packet. The base station *p*_10_ selects a reliable route using the fuzzy reasoning algorithm After performing the knowledge inference, a route sprouting tree can be illustrated in Figure 9. The different branches in the sprouting tree represent the different routes. From the sprouting tree, we can get 10 routes from the source cluster head to the base station. Comparing the truth degree of the base station, Max(0.47, 0.53, 0.41, 0.46, 0.52, 0.59, 0.58, 0.66, 0.39, 0.43) = 0.66. The larger truth degree means higher reliability of the routes. Therefore, the route 1 → 2 → 6 → 9 → 10 should be chosen,which ensures the high reliability and low packet delivery delay in communication.

### 4.3. REEMR Algorithm Analysis

REEMR is a distributed algorithm, where the nodes decide to be cluster heads according to the local neighbor nodes.

**Proposition 1** *The control overhead complexity of cluster formation in REEMR is O*(*N*)*, where N is the number of nodes.*

PROOF. At the beginning of each round, every node first broadcasts a message ADV_E to calculate the centrality and the number of its neighbor, and the maximal total ADV_E overhead is *N*. Whereafter according to the cluster head declaring time, each node transmits the message JOIN or ADV_HEAD. We assume *k* cluster heads are chosen, and then they broadcast *k* ADV_HEAD message to let the sensor nodes know about their presence. In the meantime, the other *N* − *k* cluster member nodes broadcast JOIN messages. Hence, the total message overhead is *N* + *k* + *N* − *k* = 2*N* and the control overhead complexity is *O*(*N*).

**Proposition 2** *The upper bound of the time complexity of the proposed fuzzy reasoning algorithm is O*(*nm*)*, where n is the number of places, and m is the number of transitions.*

PROOF. For a place *p_i_*, the maximum number of transitions in its postset is *m*, so the maximum computation times of the truth degree for its IRS are *m*. For *n* places, their maximum computation times are *n* × *m*. Therefore, the upper bound of the time complexity of the fuzzy reasoning algorithm is *O*(*nm*).

In REEMR, the nodes with higher residual energy, more neighbor nodes and less centrality are more likely to be elected as cluster heads. The node whose degree of reliability is less than the corresponding threshold of transition will not flood any data. Therefore the route is reliable and is not easily broken.

## 5. Performance Evaluation

We conducted several experiments to evaluate the performance of REEMR protocol and compare it to other protocols.

### 5.1. Network Models

We assume *N* sensor nodes to be uniformly distributed in an *M* × *M* field, with the following properties:

1. All the sensor nodes are stationary after the sensors are deployed.
2. All the sensor nodes are time synchronous.
3. All the sensor nodes are homogeneous and power limited, and they have exclusive identification.
4. All the sensor nodes are equipped with power control capabilities to vary their transmitted power.
5. The communication link is symmetrical. The nodes can estimate the distance between the transmitter and receiver by the received signal strength indication (RSSI).

We assume a radio hardware energy dissipation model, similar to those used in [7]. To transmit an *l*-bit message a distance *d*, the radio consumes

(5) $$E_{\mathrm{Tx}}\;\left(l,d\right)=\{\begin{array}{ll} {\mathrm{lE}}_{\mathrm{elec}}+l{ɛ}_{\mathrm{fs}}d^{2}, & d<d_{0}\\ {\mathrm{lE}}_{\mathrm{elec}}+l{ɛ}_{\mathrm{mp}}d^{4}, & d\geq d_{0} \end{array}$$

and to receive this message, the radio consumes:

(6) $$E_{\mathrm{Rx}}\;\left(l\right)=E_{\mathrm{Rx}-\mathrm{elec}}\left(l\right)=lE_{\mathrm{elec}}$$

The electronics energy, *E_elec_*, depends on factors such as the digital coding, modulation, filtering, and spreading of the signal, whereas the amplifier energy, *ɛ_fs_d*^2^ or *ɛ_mp_d*^4^, depends on the distance to the receiver and the acceptable bit-error rate.

In WSNs, the cluster heads need to aggregate and fuse the data, and the energy for data aggregation parameter is set as *E_DF_* .

### 5.2. Simulation and Analysis

To illustrate the performance of the proposed algorithm, the simulations are performed in Matlab. Each round consists of a clustering phase, multi-hop routing phase and data transmission phase. In the clustering phase, a set of cluster heads is elected and the remaining nodes become cluster members. In multi-hop routing phase, multi-hop route is set up. In the data transmission phase, each cluster member node sends a fixed amount of data to its cluster head, and each cluster head aggregates the received data and then transmits to the base station by multi-hop routing.

We firstly consider a wireless sensor network with *N* = 200 nodes randomly uniformly distributed in a 200 m *×* 200 m area. Without loss of generalization, the base station is located at [100, 250]. Assume that collisions, packet losses, and errors are dealt with at the lower layers of the protocol stack. To demonstrate the benefits of REEMR, the performance of this protocol is compared with the other typical protocols, LEACH [7], MOECS [5], CHEF [10] and PEGASIS [14], as indicated in the Section 1. The radio parameters used in our simulations are shown in Table 1. All simulation results are expressed in averages taken over 1,000 random independent experiments.

**Figure 10.**
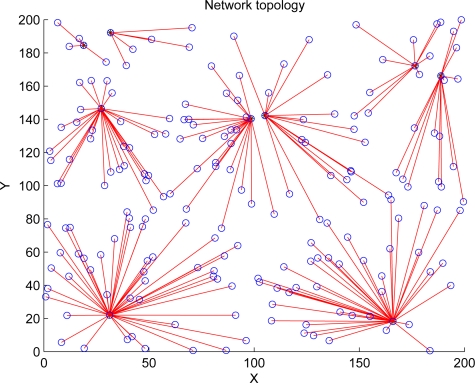
Cluster formation of LEACH.

**Figure 11.**
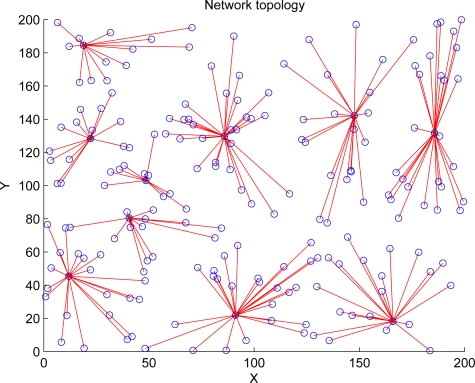
Cluster formation of REEMR.

The cluster formations of LEACH and REEMR in the same round are shown in Figures 10 and 11. In LEACH, some cluster heads are close each other, and cluster member nodes in each cluster are uneven because LEACH depends only on the probability model to select cluster heads. On the other hand, as REEMR considers the node residual energy and local topology, the cluster heads in REEMR are more even than those in LEACH.

The network lifetime is the most important performance metric for WSNs. In [24], the network lifetime is defined as the time until the first node fails or runs out of energy. According to the definition, we can define the network lifetime as the round until the first node runs out of energy. To further evaluate the REEMR’s performance with respect to scalability, we employ two different network sizes, namely *N* = 200 and *N* = 400.

**Figure 12.**
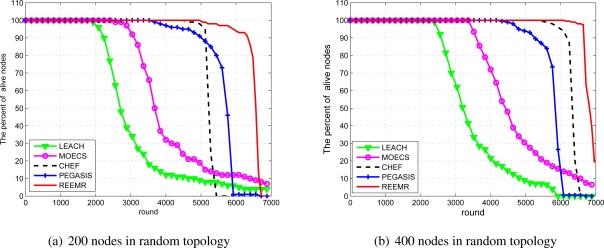
The network lifetime in rounds for random topology.

Figure 12 demonstrates that the network lifetime of REEMR is longer than that of the other protocols, and the scalability of REEMR is better than of the other protocols. In REEMR, the first node death occurs after 4,999 rounds and 6,081 rounds for network size of 200 and 400 nodes, respectively. Under the first node death criterion REEMR extends the network lifetime approximately 13% compared to CHEF, 42% compared to PEGASIS, 77% compared to MOECS, and 156% compared to LEACH. This is because REEMR considers the residual energy, the number of neighbors, and the centrality that influence energy consumption to elect cluster heads. Furthermore, cluster heads construct a reliable multi-hop route which is not easily broken, but also reduces the control overhead. In REEMR, all the sensor nodes can consume the energy evenly, and so the energy consumption of the networks is balanced.

As LEACH randomly chooses the cluster heads and their distribution is not uniform, some cluster heads may have too many cluster member nodes. Therefore, these cluster heads may consume much energy and die too soon. MOECS considers multiple parameters such as the distance of a node to the cluster head and residual energy to select cluster heads, which help sensor nodes achieve balanced energy dissipation to prolong the network lifetime. CHEF considers the energy and local distance to choose the optimal cluster heads, which are uniformly deployed in the networks. Consequently CHEF further prolongs the lifetime of the networks than LEACH. In PEGASIS, the distance of the neighbor nodes is less than that of the cluster members and the cluster heads in LEACH, and its head only receives the data of two nodes. Consequently, PEGASIS achieves higher performance than LEACH.

**Figure 13.**
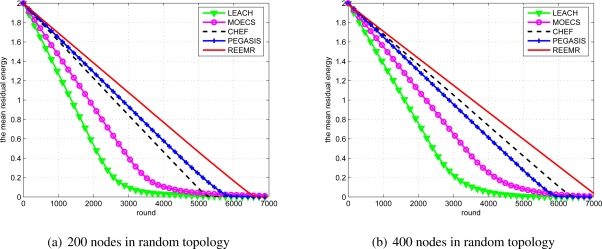
Mean residual energy of LEACH, MOECS, CHEF, PEGASIS, and REEMR.

**Figure 14.**
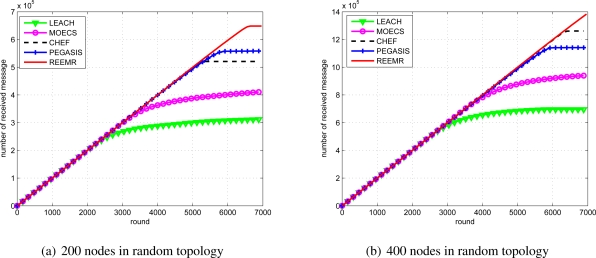
Total number of data messages received at the base station.

The residual energy of the network also provides an estimate of the network lifetime. Figure 13 illustrates the mean residual energy of the network. It can be observed that the mean residual energy of the node in the case of REEMR is higher than that of the other protocols. Hence, the network lifetime under REEMR protocol is enhanced compared to LEACH, MOECS, CHEF and PEGASIS. These results corroborate the results presented in Figure 12. Figure 14 illustrates the received data at the base station. Under the first node death criterion, the received data using REEMR is approximately 11% higher than CHEF, 45% higher than PEGASIS, 68% higher than MOECS and 153% higher than LEACH.

## 6. Conclusions

Routing algorithms have been well considered as one of the effective solutions to enhance energy efficiency and scalability of wireless sensor networks. In this paper, a reliable energy-efficient multi-level routing algorithm for wireless sensor networks is proposed, which considers residual energy, number of neighbors and centrality for cluster formation which are critical for well-balanced energy dissipation of the network. REEMR employs fuzzy Petri nets to choose cluster heads and construct multi-hop routing among cluster heads by fuzzy reasoning algorithm.

Simulation results demonstrate that REEMR achieves significant energy savings and prolongs network lifetime when compared to LEACH, MOECS, CHEF and PEGASIS. Multiple parameters involved in the cluster formation process for REEMR help sensor nodes dissipate their energy at a much more balanced rate as compared to other protocols.

## Figures and Tables

**Table 1.** Simulation parameters.

**Parameter**	**Value**
Initial energy per node	2 J
*E_elec_*	50 nJ/bit
*ε_fs_*	10 pJ/(bit·*m*^2^)
*ε_mp_*	0.0013 pJ/(bit·*m*^4^)
*d*_0_	87 m
*E_DF_*	5 nJ/(bit· signal)
Size of a data packet	500 bits

## References

[1] Akyildiz LF, Su W, Sankarasubramaniam Y, Cayirci E (2002). Wireless sensor networks: A survey. Comput. Netw.

[2] Yick J, Mukherjee B, Ghosal D (2008). Wireless sensor network survey. Comput. Netw.

[3] Heinzelman WB, Chandrakasan AP, Balakrishnan H (2002). An application specific protocol architecture for wireless microsensor networks. IEEE Trans. Wirel. Commun.

[4] Abbasi AA, Younis M (2007). A survey on clustering algorithms for wireless sensor networks. Comput. Commun.

[5] Dimokas N, Katsaros D, Manolopoulos Y (2010). Energy-efficient distributed clustering in wireless sensor networks. J. Parall. Distrib. Comput.

[6] Natsheh E (2008). A survey on fuzzy reasoning applications for routing protocols in wireless *ad hoc* Networks. IJBDCN.

[7] Heinzelman WB, Chandrakasan AP, Balakrishnan H Energy-efficient communication protocol for wireless microsensor networks.

[8] Muruganathan SD, Ma DCF, Bhasin RI, Fapojuwo AO (2005). A centralized energy-efficient routing protocol for wireless sensor networks. IEEE Radio Commun.

[9] Gupta I, Riordan D, Sampalli S Cluster-head election using fuzzy logic for wireless sensor networks.

[10] Kim JM, Park SH, Han YJ, Chung TM CHEF: Cluster head election mechanism using fuzzy logic in wireless sensor networks.

[11] Haider T, Yusuf M (2009). A fuzzy approach to energy optimized routing for wireless sensor networks. IAJIT.

[12] Tashtoush M, Okour MA Fuzzy self-clustering for wireless sensor networks.

[13] Liang Q Fault-tolerant and energy efficient wireless sensor networks a cross-layer approach.

[14] Lindsey S, Raghavendra CS PEGASIS: Power-efficient gathering in sensor information systems.

[15] Younis O, Fahmy S (2004). HEED: A hybrid, energy-efficient, distributed clustering approach for *ad hoc* sensor networks. IEEE Trans. Mob. Comput.

[16] Qing L, Zhu Q, Wang M (2006). Design of a distributed energy-efficient clustering algorithm for heterogeneous wireless sensor networks. Comput. Commun.

[17] Ye M, Li C, Chen G, Wu J (2006). An energy efficient clustering scheme in wireless sensor networks. Ad Hoc Sens. Wirel. Netw.

[18] Chamam A, Pierre S (2010). A distributed energy-efficient clustering protocol for wireless sensor networks. Comput. Electr. Eng.

[19] Aslam N, Phillips W, Robertson W, Sivakumar S (2009). A multi-criterion optimization technique for energy efficient cluster formation in wireless sensor networks. Inform Fusion.

[20] Chen SM, Ke JS, Chang JF (1990). Knowledge representation using fuzzy Petri nets. IEEE Trans. Knowl. Data Eng.

[21] Gao MM, Zhou MC, Huang XG, Wu ZM (2003). Fuzzy reasoning Petri nets. IEEE Trans. Syst. Man Cybern. A Syst. Hum.

[22] Lao L, Cui JH (2006). Reducing multicast traffic load for cellular networks using *ad hoc* networks. IEEE Trans. Veh. Technol.

[23] Chiang TC, Tai CF, Hou TW (2009). A knowledge-based inference multicast protocol using adaptive fuzzy Petri nets. Expert Syst. Appl.

[24] Karl H, Willing A (2005). Protocols and Architectures for Wireless Sensor Networks.

